# First Detection and Tunneling Time of a Quantum Walk

**DOI:** 10.3390/e25081231

**Published:** 2023-08-18

**Authors:** Zhenbo Ni, Yujun Zheng

**Affiliations:** 1 School of Physics, Shandong University, Jinan 250100, China; realmilan@126.com; 2 Department of Physics, Bar Ilan University, Ramat-Gan 52900, Israel

**Keywords:** continuous-time quantum walk, first detection time

## Abstract

We consider the first detection problem for a one-dimensional quantum walk with repeated local measurements. Employing the stroboscopic projective measurement protocol and the renewal equation, we study the effect of tunneling on the detection time. Specifically, we study the continuous-time quantum walk on an infinite tight-binding lattice for two typical situations with physical reality. The first is the case of a quantum walk in the absence of tunneling with a Gaussian initial state. The second is the case where a barrier is added to the system. It is shown that the transition of the decay behavior of the first detection probability can be observed by modifying the initial condition, and in the presence of a tunneling barrier, the particle can be detected earlier than the impurity-free lattice. This suggests that the evolution of the walker is expedited when it tunnels through the barrier under repeated measurement. The first detection tunneling time is introduced to investigate the tunneling time of the quantum walk. In addition, we analyze the critical transitive point by deriving an asymptotic formula.

## 1. Introduction

Nowadays, quantum walk (QW), a counterpart of the classical random walk, plays a key role in many quantum problems [1,2,3,4,5,6,7,8,9,10]. The quantum walk, introduced by Aharonov et al. [11], can be realized in two distinct ways: the continuous-time quantum walk (CTQW) [12,13,14] and the discrete-time quantum walk (DTQW) [4,15,16,17]. In the continuous case, the evolution of a particle is described by the Schrödinger equation and the Hamiltonian of a system is related to the transition matrix of graphs which represents the transition rates of the corresponding continuous-time random walk. In the discrete-time scenario, a unitary quantum coin operator is used to determine the direction of the particle’s movement (e.g., left or right in a one-dimension lattice system), in addition to a shift operator which describes the movement of the quantum walker. Studies of quantum walks have demonstrated that these two approaches display the same qualitative behaviors [4,14,18]. The quantum algorithm based on a CTQW is exponentially faster than the classical algorithm of a classical random walk for the “glued trees” graph [14]. The quantum search algorithm based on the DTQW has a quadratic speed-up $O(\sqrt{N})$ on a database of *N* items [4]. Recently, different experimental implementations of quantum walks have been performed by photons in waveguide, nuclear magnetic resonance, and trapped atom systems [19,20,21].

In consideration of the acceleration property of the quantum walk in quantum algorithms, quantum walks have drawn considerable attention, and much progress has been achieved on first detection problems [22,23,24,25,26,27,28,29,30,31,32,33]. Krovi et al. defined the hitting time by analogy to a classical random walk for a quantum walk on a hypercube [23,25]. Several studies [27,29,34] have derived a quantum renewal equation for the first detection time which is similar to the classical renewal equation and discussed dark states, uncertainty principles, and transition times on various graphs. For example, in a closed ring system with a repeated measurement interval τ the mean first detection return time is 〈n〉τ, with 〈n〉 provided by the number of distinct energy levels of the system. Thiel et al. studied the return and arrival problems of the first detection probability on the infinite line, where the behavior is highly sensitive to the distance between the detection site and the initial localized particle. Beyond all expectations, the total detection probability becomes independent of the distance when it is large [27,32]. Considerable work on first detection probability in quantum walks has investigated a single particle in systems with an absence of defects. Unlike DTQW, which can perform a biased walk, a CTQW evolves as a ballistic spread to both sides. Hence, we would like to adopt a Gaussian wave packet to dominate the direction and the velocity of the initial condition. Moreover, the systems realizing the quantum walk may comprise defects or disorder in realistic experimental realization. Thus, quantum walks in impure systems should be addressed as well.

In this paper, we consider a quantum walk on a one-dimension lattice under tight binding approximation. We investigate the effect of the initial Gaussian wavepacket with some initial mean momentum, rather than the delta-function localized at a given site, as considered previously [32]. We find that the long time behavior of the first detection probability with a broad initial state decays monotonically, contrary to the case of the quantum point source, where it decays with superimposed oscillations. We derive an asymptotic formula to describe the relation between the width of the initial wave packet and the oscillation of the first detection probability. The initial superposition lessens the influence of the sampling time on the total detection probability. When the wave packet is wide, the exceptional sampling time remains, and the total detection probability is a smooth function relative to the sampling time rather than oscillating. This provides more options in terms of the sampling time used to detect the particle.

**Figure 1:**
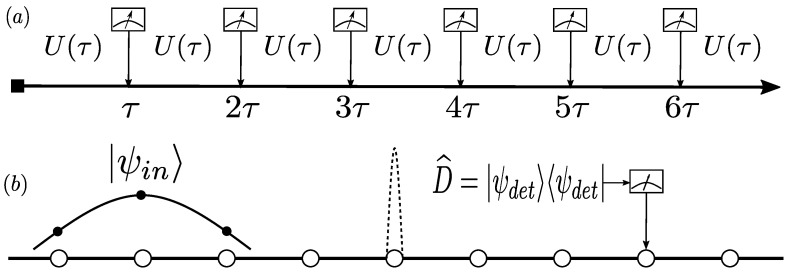
(**a**) The stroboscopic detection protocol. The particle propagates freely between two adjacent measurements, and the detection time interval is τ. (**b**) The lattice system for the quantum walk. The initial condition $|\psi_{in}\rangle$ is a wave packet, and we measure whether the particles arrive at the destination by a projective operator $\hat{D}=|\psi_{det}\rangle \langle \psi_{det}|$. The dashed line denotes a barrier located at a lattice. The cases with and without a barrier are discussed in Section 2.2 and Section 2.3, respectively.

We then study the system in the presence of a tunneling center on one lattice point, that is, we consider the effects of a localized impurity on the first detection probability. As Figure 1 shows, the impurity is modeled with a delta-like potential barrier, with a height ϵ (units energy). We consider an incoming initial Gaussian wave packet with a mean momentum traveling from left to right, setting the detector on the right of the barrier. By employing the stroboscopic detection protocol [27], we study the first detection probability and the total probability with different initial waves. In addition, we introduce the tunneling time in the first detection time scenario to present the tunneling time and multiple-defects tunneling. We find that the speed inside the barrier is faster than the speed outside the barrier, that is, the evolution can be accelerated by the barriers but the detection probability becomes lower. For the first detection case, the measurements play a crucial role because of the backfire and modify the unitary evolution before the final successful detection.

The rest of this paper is structured as follows. In Section 2, we introduce the basic theoretical framework of the CTQW and the measurement protocol. The first detection probability of the Gaussian wave quantum walk is derived. The quantum walk in the system with a barrier is provided in this section as well. Section 3 shows the numerical results of the first detection problems in the presence and absence of tunneling. In Section 4, we present the asymptotic result of the first detection probability. Section 5 closes the paper with our concluding remarks.

## 2. Theoretical Framework

### 2.1. Stroboscopic Protocol

We first recall the basics of the stroboscopic detection protocol. The CTQW, a quantum analogy of the classical continuous-time random walk, replaces the classical vector of probabilities with a vector of probability amplitudes (a state vector) and the transition matrix with a unitary matrix. We consider a quantum walk on a one-dimension tight-binding model with hopping to the nearest neighbor sites only. Its propagation can be described by the Schrödinger equation, and the unitary U(t) is provided by the following equation (we set ħ=1):

(1) $$U\left(t\right)=e^{-iHt}.$$

The Hamiltonian *H* is defined as [1,8]

(2) $$\begin{array}{c} H_{jj^{\prime }}=\left\{\begin{array}{cc} -\gamma , & \mathrm{if}\;j=j^{\prime }\pm 1\;,\\ 0, & \mathrm{otherwise}\;, \end{array}\right. \end{array}$$

where the integer indices *j* and $j^{\prime }$ denote the lattice coordinates and γ is the hopping rate. The state of the system in the absence of repeated measurements at time *t* is

(3) $$|\psi (t)\rangle =U\left(t\right)|\psi_{in}\rangle ,$$

with the initial state $|\psi_{in}\rangle$.

We define the projection operator, corresponding to a measurement to detect the particle, as follows:

(4) $$\hat{D}=|\psi_{det}\rangle \langle \psi_{det}|.$$

The probability of detecting the particle is

(5) $$p_{1}={\left|\langle \psi_{det}|\psi \rangle \right|}^{2}=\langle \psi |\psi_{det}\rangle \langle \psi_{det}|\psi \rangle =\langle \psi |\hat{D}|\psi \rangle ;$$

correspondingly, the probability of non-detecting the particle is $p^{\prime }=1-p_{1}$. Based on the projection measurement postulates of quantum mechanics [35], the state of the system after a failed detection is immediately the normalized projection, namely,

(6) $$|\psi^{\prime }\rangle =\frac{(1-\hat{D})|\psi \rangle }{\sqrt{1-p_{1}}}.$$

The primes in $p^{\prime }$ and $|\psi^{\prime }\rangle$ represent other variables rather than the derivative. As shown in Figure 1, we consider the stroboscopic measurement protocol [24,27,29]; a sequence of measurements is described by employing the same projector with the time interval τ between measurements. The probability of detection after the preceding (n−1) measurements has failed is

(7) $$p_{n}=\frac{|\langle \psi_{det}|{\left[U\left(\tau \right)\left(1-\hat{D}\right)\right]}^{n-1}U\left(\tau \right)|\psi_{in}{\rangle |}^{2}}{\prod_{m=1}^{n-1}\left(1-p_{m}\right)}.$$

Hence, the probability of first detection after *n* attempts is [24,27]

(8) $$\begin{array}{cc} F_{n} & =\left(1-p_{1}\right)\left(1-p_{2}\right)\left(1-p_{3}\right)\cdots \left(1-p_{n-1}\right)p_{n}\\ \; & =|\langle \psi_{det}|{\left[U\left(\tau \right)\left(1-\hat{D}\right)\right]}^{n-1}U\left(\tau \right)|\psi_{in}{\rangle |}^{2}. \end{array}$$

It is convenient to define the amplitude of the first detection as [24,27,32]

(9) $$\varphi_{n}=\langle \psi_{det}|{\left[U\left(\tau \right)\left(1-\hat{D}\right)\right]}^{n-1}U\left(\tau \right)|\psi_{in}\rangle ,$$

and we can show that $\varphi_{n}$ satisfies the following quantum renewal equation [27]:

(10) $$\varphi_{n}=\langle \psi_{det}\left|U\left(n\tau \right)\right|\psi_{in}\rangle -\sum_{m=1}^{n-1}\varphi_{m}\langle \psi_{det}\left|U\left[\left(n-m\right)\tau \right]\right|\psi_{det}\rangle .$$

In Equation (10), the first term describes the direct transition from the initial location to the detector, and the second term the paths that arrive at the detector at the $m^{\mathrm{th}}$ measurement and then return in the remaining “time” (n−m). Equation (10) can be solved with a generating function technique and convolution theorem [36].

Correspondingly, the total probability that the particle can be detected is

(11) $$P_{tot}=\sum_{n}F_{n}={\left|\varphi_{n}\right|}^{2}.$$

To solve the quantum renewal equation Equation (10), as a useful technique for dealing with discrete random events in time, we introduce the generating function here, which may be considered as the discrete analogue of the Laplace transform [8,29,37,38]:

(12) $$\hat{\varphi }\left(z\right)=\sum_{n=1}^{\infty }z^{n}\varphi_{n},$$

where *z* is an auxiliary variable. Using the convoluting quantum renewal equation of Equation (10), we can obtain [27,29]

(13) $$\hat{\varphi }\left(z\right)=\frac{\langle \psi_{det}|\hat{U}\left(z\right)|\psi_{in}\rangle }{1+\langle \psi_{det}|\hat{U}\left(z\right)|\psi_{det}\rangle },$$

where

(14) $$\hat{U}\left(z\right)=\sum_{n=1}^{\infty }z^{n}U^{n}\left(\tau \right)=\sum_{n=1}^{\infty }z^{n}e^{-iHn\tau }.$$

After $\hat{\varphi }\left(z\right)$ has been obtained, we can find $\varphi_{n}$ by the inverse transformation

(15) $$\varphi_{n}=\frac{1}{n!}\frac{d^{n}}{dz^{n}}\hat{\varphi }{\left(z\right)|}_{z=0},$$

or by

(16) $$\varphi_{n}=\frac{1}{2\pi i}\oint_{C}\hat{\varphi }\left(z\right)z^{-n-1}dz,$$

where *C* is a counterclockwise contour around the origin and is within the radius of convergence of the generating function $\hat{\varphi }\left(z\right)$.

### 2.2. Gaussian Wave Packet Quantum Walk

For the tight-binding model, after considering Equation (2), we have the following Hamiltonian [27,39]:

(17) $$H=-\gamma \sum_{j}(|j\rangle \langle j+1|+|j+1\rangle \langle j|),$$

where |j〉 represents the state localized at site *j*. In addition, |j〉 is part of a complete orthonormalized basis set, namely, $\langle j^{\prime }|j\rangle =\delta_{j^{\prime }j}$ and $\sum_{j}\left|j\rangle \langle j\right|=1$. The set of sites {j} forms an infinite lattice. We assume the hopping rate γ=1 in the remainder of this work for the sake of simplicity. The eigenvalues of the tight-binding model Hamiltonian of Equation (17) are [27,32,39]

(18) $$E_{k}=-2\cos k$$

with eigenstates

(19) $$|E_{k}\rangle =\frac{1}{\sqrt{2\pi }}\sum_{j}e^{ikj}|j\rangle ,$$

where the wave vector is −π≤k≤π in the first Brillouin zone.

To demonstrate the quantum walk of a Gaussian wave packet, we suppose the normalized initial wave function can be expressed as

(20) $$|\psi_{in}\rangle =\sum_{j}c_{j}|j\rangle ,$$

where, after supposing the mean position of the wave packet to be $j_{c}$ and its carrying lattice momentum to be $k_{0}$, the weights can be written as [40]

(21) $$\begin{array}{c} c_{j}=\frac{1}{\sqrt{A}}e^{-ik_{0}j}e^{-\frac{{\left(j-j_{c}\right)}^{2}}{4\sigma^{2}}} \end{array}$$

with the normalized constant $A=\sum_{j}e^{-\frac{j^{2}}{2\sigma^{2}}}$, and σ as the width of the Gaussian wave packet.

By employing the fact of the matrix elements of the propagator [27,32]

(22) $$\begin{array}{cc} \langle j^{\prime }|U\left(n\tau \right)|j\rangle & =\frac{1}{2\pi }\int_{-\pi }^{\pi }e^{ik(j^{\prime }-j)}e^{-in\tau E_{k}/\hbar }dk\\ \; & =i^{j^{\prime }-j}J_{j^{\prime }-j}\left(2n\tau \right), \end{array}$$

we obtain the generating function of the first detection amplitude for a Gaussian wave quantum walk

(23) $$\hat{\varphi }\left(z\right)=\frac{\sum_{n=1}^{\infty }z^{n}\sum_{j=-\infty }^{\infty }i^{j_{d}-j}c_{j}J_{j_{d}-j}\left(2n\tau \right)}{1+\sum_{n=1}^{\infty }z^{n}J_{0}\left(2n\tau \right)},$$

where $i=\sqrt{-1}$ as usual and $J_{\mu }\left(x\right)$ is the Bessel function of the first kind of order μ. The generating function of the first detection amplitude of the Gaussian wave quantum walk is the sum of the packet’s components with different weights, which are determined by the initial Gaussian wave packet. This is clearly the superposition principle at work. The order of the Bessel function in the numerator is only related to the distance between the detection site and the initial position of every component. Using Equations (11) and (15), we can obtain the first detection probability of the Gaussian wave packet quantum walk.

### 2.3. Quantum Walk with Tunneling

In this subsection, we consider the tight-binding model in which one of the diagonal matrix elements is equal to ϵ; as a consequence, the Hamiltonian can be defined as [39,41]

(24) $$\begin{array}{c} H=H_{0}+H_{1}=-\sum_{j}(|j\rangle \langle j+1|+|j+1\rangle \langle j|)+\epsilon |0\rangle \langle 0|, \end{array}$$

where the barrier is located at site j=0. In this paper, we assume that the magnitude of the peculiar ingredient ϵ is positive, meaning that the quantum walker may reflect or transmit when it impinges the barrier. In order to calculate the first detection probability, it is necessary to obtain the propagator. Using the theory of the Green function [41], which is shown in Appendix A.1 for a tight-binding Hamiltonian with a single impurity, we can obtain the matrix elements of the propagator as follows:

(25) $$\begin{array}{cc} \langle j^{\prime }|U\left(n\tau \right)|j\rangle & =i^{j^{\prime }-j}J_{j^{\prime }-j}\left(2n\tau \right)+e^{-in\tau \sqrt{\epsilon^{2}+4}}\frac{2^{-|j^{\prime }\left|-|j\right|}\left|\epsilon \right|{\left(\epsilon -\sqrt{\epsilon^{2}+4}\right)}^{|j^{\prime }\left|+|j\right|}}{\sqrt{\epsilon^{2}+4}}+\\ \; & \frac{1}{\pi }\int_{0}^{\pi }dk\left(e^{i2n\tau \cos k}\frac{i\epsilon e^{-ik|j|}}{2\sin k-i\epsilon }\cos kj^{\prime }+\frac{-i\epsilon e^{ik|j^{\prime }|}}{2\sin k+i\epsilon }\cos kj+\frac{\epsilon^{2}e^{ik(|j^{\prime }|-|j|)}}{4\sin^{2}k+\epsilon^{2}}\right). \end{array}$$

Equation (25) is composed of the bound state ingredient and the scattered state ingredient. Employing the definition of the generation function Equation (13) and the inverse transformation Equation (15), we can obtain the first detection probability $F_{n}$. We calculated the integral in Equation (25) and the series of the inverse transformation using *Mathematica*.

## 3. Numerical Results

In this section, we show the numerical results of the models discussed in Section 2. We consider the quantum walk with a Gaussian wave packet which is centered at site $j_{c}$ as an initial condition. The detection state is $|\psi_{det}\rangle =|j_{d}\rangle$. In addition, we investigate the tunneling time based on the first detection probability with an impurity.

### 3.1. Gaussian Wave Packet Quantum Walk with ϵ=0

**Figure 2:**
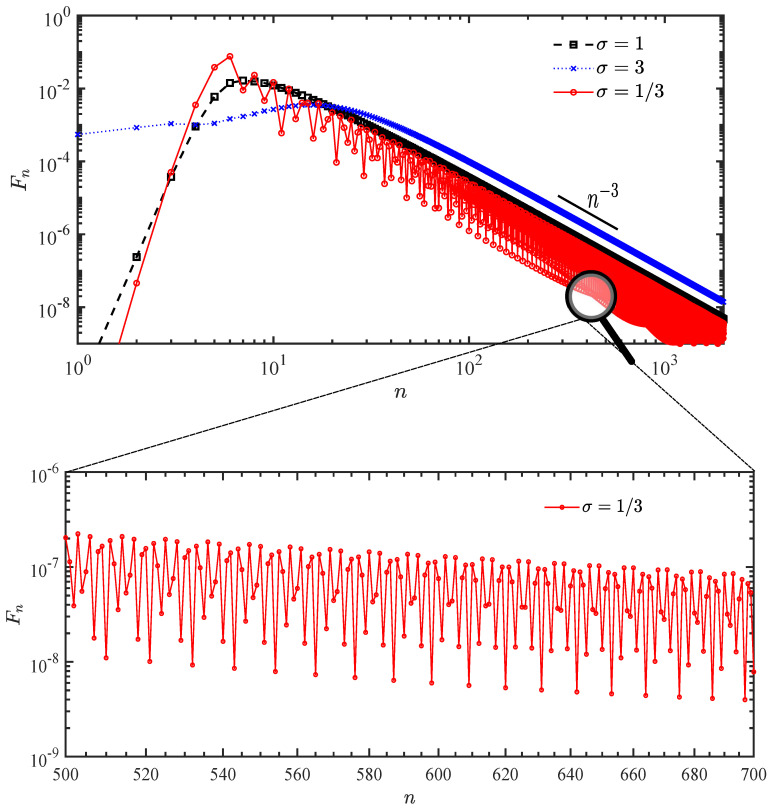
The probabilities of the first detection for different initial conditions (log−log). The initial condition is a Gaussian wave packet which centered at $j_{c}=-10$, and the detection state is |0〉. The sampling time is τ=1 and the momentum $k_{0}=0$. The circles, squares, and crosses numerically obtained by Equation (23) represent the results of the width of the Gaussian wave packet σ=1/3,1,3, respectively. Generally, $F_{n}\propto n^{-3}$ with superimposed oscillations, the latter vanish when the initial width of the wave packet σ is large.

We consider the first detection probability distribution and the total detection probability in this subsection. Figure 2 demonstrates the first detection probability with different widths σ of the Gaussian initial wave packet; the circles, squares, and crosses correspond to the results of σ=1/3,1,3, respectively. When the width of the Gaussian wave packet σ is small, the behavior of the probability $F_{n}$ is similar to the case of a single source exhibiting a power-law decay superimposed with oscillations [27,32]. By increasing the width of the initial wave packet, we thereby reduce its spread in terms of its momentum, and the decay of the first detection probability changes from oscillatory to monotonic. In contrast to the classical continuous-time random walk, although both decay monotonically, the rates of power-law decay differ, with values of −3 (quantum) and −3/2 (classical), respectively [32]. In order to depict the velocity of the walker, we employ the incident time $t_{in}=n_{in}\tau$ when the maximal peak of the first detected probabilities appears. When the initial condition is a localized state, we find that the incident time is $n_{in}=\left|j_{d}-j_{c}\right|/v_{g}\tau$, where $v_{g}$ denotes the maximum group velocity and $v_{g}=\max\left|E_{k}^{\prime }\right|=2$ [27,32]. As shown in Figure 2, a similar behavior is found for σ=1/3 (circles). For the wide wave packet, the transport time $t_{in}$ is proportional to the initial fluctuation of the momentum, i.e., the width of the wave packet in wave vector space can be obtained by the Fourier transformation or Heisenberg’s uncertainty principle, and is no longer the ballistic spread exhibited by an initial localized state.

**Figure 3:**
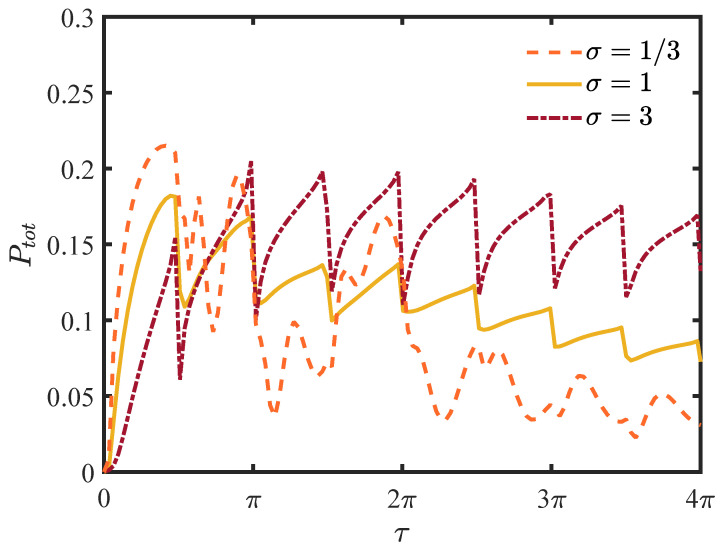
The plot of the total detection probability. The horizontal ordinate τ is the sampling time and the vertical coordinate represents the total probability given by Equation (11). For small τ, we have the Zeno limit, namely, the particle cannot be detected at all. The non-analytical behaviour at the sampling time provided by Equation (26) is visible with a finite width of the packet.

In Figure 3, we display the total detection probability for different widths of wave packets. The total probability is less than unity in this infinite lattice system because there is a portion of the wave function propagating in the direction opposite to that of the detection site. This is very different from the classical case, in which the motion is diffusive and recurrent, and the walker will eventually be found. The probability in the vicinity of the origin of the sampling time remains nearly zero, the evolution of walker being impeded by the high frequency repeated measurements, that is, the Zeno effect [27,42,43]. The exceptional sampling time τ satisfies the relation [27]

(26) ΔEτ=2πm,

where ΔE=4 is the width of the energy band in Equation (18), *m* is an integer, and the derivative of the probability is not continuous. There is a jump in the vicinity of the exceptional sampling time. Compared to the single localized initial condition in [32] or the σ=1/3 scenario in Figure 3, the total probability increases monotonically between the adjacent critical sampling times. As the width widens, the highest peak of the total detection probability is not always at τ=π/2, and the optimal sampling time is not unique.

### 3.2. Quantum Walk with an Impurity

In this subsection, we present the numerical results of the first detection probability and the total detection probability of the system with a single impurity.

**Figure 4:**
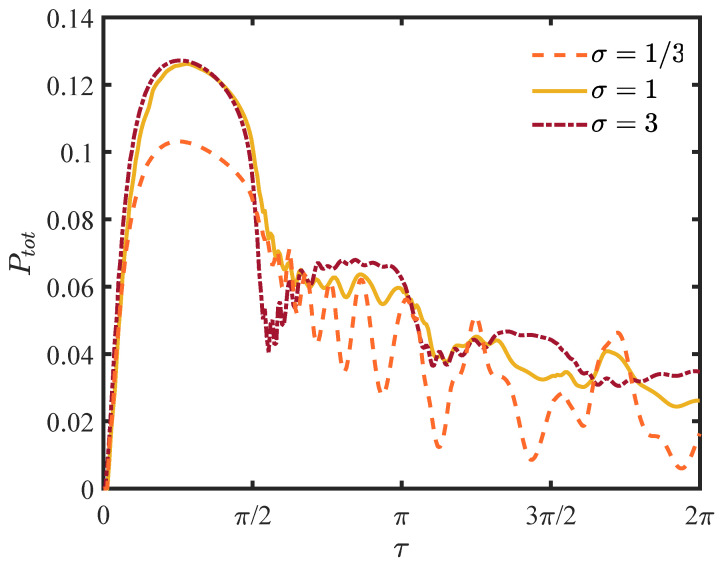
The total detection probability for different widths of the wave packet. The initial condition is a Gaussian wave packet centered at $j_{c}=-10$. The detection site is located at $j_{d}=20$, and the magnitude of the barrier is ϵ=2.

First, we consider the relationship between the sampling time and the total detection probability. As shown in Figure 4, the highest peak appears immediately after raising the sampling time, and the sampling time of the peak is much smaller than the case without the barrier, namely $\tau =\frac{\pi }{2}$. In presence of a barrier, the pedestrian will pass through the barrier or be reflected by the barrier. Except for the left-moving initial wave, the reflected waves have no contributions to the total probability. When the sampling time τ>π/2, the total probability oscillates without monotonic behaviour and there are no conspicuous peaks.

**Figure 5:**
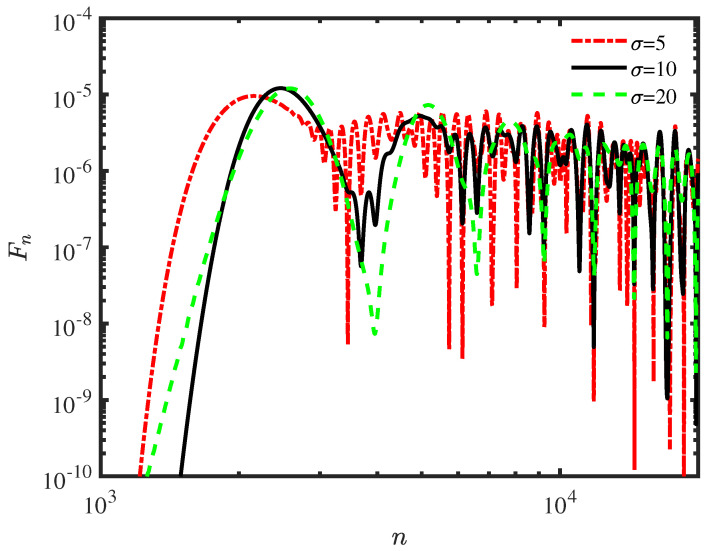
The probability distribution $F_{n}$ of first detection in the *n*th attempt for the different widths of the wave packet (log−log). The initial condition is a Gaussian wave packet centered at $j_{c}=-100$, the wave vector is $k_{0}=\pi /8$, and the sampling time is τ=0.1. We measured the particles at $j_{d}=100$, and the magnitude of the barrier is ϵ=2.

Figure 5 shows the probability distribution $F_{n}$ as a function of the attempted number of first detection for the transition problem in the presence of a barrier located at site j=0. The behaviors of σ=1,3, which are not shown in this figure, are similar to the case σ=5 with oscillations. In order to show different behaviors, the other two parameters are selected for presentation in Figure 5. The transport velocity is same as in the impurity-free case, that is, the wider packet propagates faster. There are many peaks resulting from the barrier and the successive measurements, and the different components of wave packet cross the barrier with different probabilities. The probability of wide wave packet decays monotonously between the adjacent peaks in comparison to the narrow initial condition. Because the narrow initial wave contains more components of wavenumbers, there is more interference with other components after passing the barrier.

### 3.3. Tunneling Time on First Detection Probability

In the presence of a barrier, the time required for a wave packet to traverse the barrier is a well-studied problem, though not free of controversies [14,44,45,46,47,48,49,50,51,52,53,54]. The analysis of tunneling time is currently debated because of the unusual and subtle role that time plays in quantum mechanics. In the community of traditional quantum mechanics, time is thought of as a nonquantum ingredient of quantum mechanics. Previously, the tunneling time has been defined in different ways, such as phase time (or group delay) [49,50,51], dwell time [51,52,53], and Larmor time [53,54].

Here, we introduce the first detection tunneling time, defined with the aforementioned stroboscopic protocol, in analogy with the phase time, which can be calculated theoretically by the frequency derivative of the transmission phase change or measured experimentally at the time instant when the peak of a tunneling wave packet impinges and transmits the barrier.

Corresponding to the phase time, we can utilize the most probable first detection time to calculate the tunneling time; we measure the walker at the detected site, rather than the boundary of the barrier. Replacing the single localized initial condition with a broad wave packet $|\psi_{in}\rangle$ that is sharply peaked in the momentum space, we represent a particle with momentum $p=\hbar k_{0}$ and velocity $v=\hbar k_{0}/m$. The distribution can be written as

(27) $$|\psi_{in}\rangle =\frac{1}{\sqrt{A}}e^{ik_{0}\left(j-j_{c}\right)}e^{-\frac{{\left(j-j_{c}\right)}^{2}}{4\sigma^{2}}},$$

where the center of the wave packet is located at $\langle j\rangle =j_{c}$ and the width is Δj=σ. The initial wave packet in the momentum representation is the Fourier transform of the position representation, that is, the wave function in the momentum representation is Gaussian as well. The corresponding standard deviation is Δk=1/σ, and the average momentum is $\langle k\rangle =k_{0}$.

**Figure 6:**
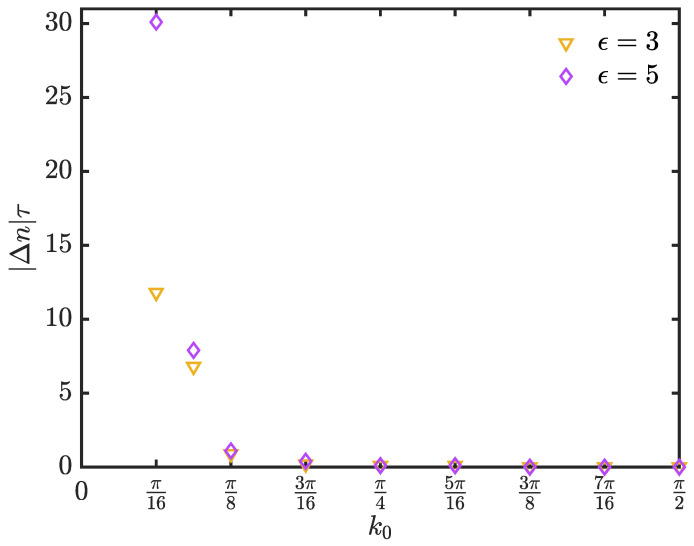
The tunneling time of a quantum walk as a function of $k_{0}$. The wide Gaussian wave packet (σ=20) is centered at $j_{c}=-100$, the magnitudes of the barrier are ϵ=3, corresponding to the orange triangles, and ϵ=5, corresponding to the purple diamonds, and the sampling time τ=0.1. We measure the walker at $j_{d}=100$. The tunneling time is provided by Equation (28).

Based on the above considerations, we can investigate the tunneling time by comparing the incident time of the barrier case to the impurity-free system. Thus, the tunneling time is provided by

(28) $$t_{tunnel}=|\Delta n\tau |=|n_{in}^{free}-n_{in}^{barrier}|\tau ,$$

where $n_{in}^{free}$ is the incident time in the absence of a barrier and $n_{in}^{barrier}$ denotes the incident time in the presence of the barrier. As demonstrated in Figure 6, we choose a wide initial wave packet to ensure that the wave vector spread of the wave packet is narrow, and we can obtain the tunneling time. A walker carrying more momentum takes the same time to reach the detection site as one in the circumstance with no barrier. For a walker with less momentum, the peak of the tunneling wave packet arrives at the detection site earlier than normal free propagation. However, this seemingly anomalous behavior does not mean a violation of Einstein causality. The so-called “phase time” cannot be regarded as the velocity of the propagation of the tunneling wave, as it arises from the head start of the initial broad wave.

**Figure 7:**
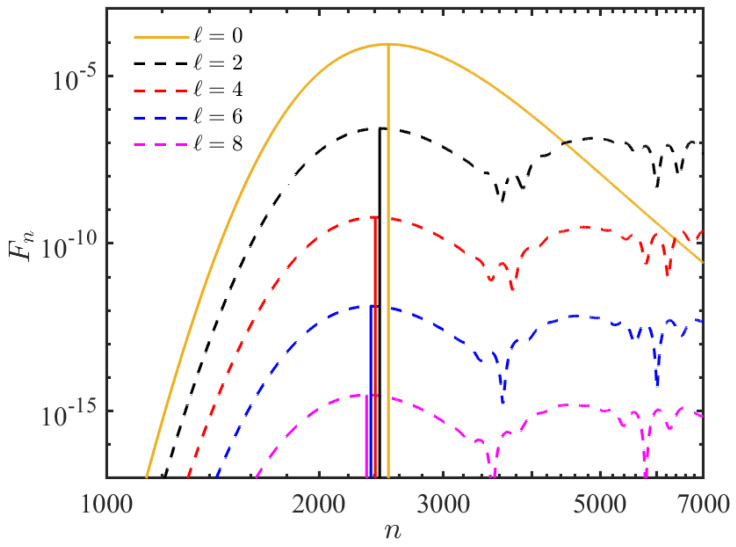
The tunneling time of a quantum walk as a function of the number of the defects, denoted by *ℓ* (log−log). The parameters of the simulation were as follows: the spread of the initial wave packet was 10, the distance between the detector and the starting point was 200, the projective measurement was operated at $j_{d}=100$, and the magnitude of the defects was 3.

Furthermore, we can extend the tunneling problem to multiple defects by simulating the first detection problem of the quantum walk. In this multiple defects scenario, the system can be expressed as

(29) $$H=-\sum_{j}(|j\rangle \langle j+1|+|j+1\rangle \langle j|)+\sum_{ȷ=0}^{\ell -1}\epsilon |ȷ\rangle \langle ȷ|,$$

where *ȷ* is the location of the defect and *ℓ* is interpreted as the spread of the barrier. The probability of the walker being detected at a specific point for the first time is determined by the Schrödinger equation and the quantum renewal equation. As shown in Figure 7, the first detection probabilities are plotted for a variety of values of *ℓ*. Using the same method, we compare the peak of the first detection probability distribution. For the scenario with multiple defects, it takes more time for the walker to cross the barrier. However, after the walker is successfully detected, the mean first passage time is less than with fewer defects. In a sense, the defects give rise to an acceleration of the evolution, that is, the speed inside the barrier is faster than the speed outside the barrier. In the meantime, the length and strength of barriers enhance the acceleration effect. However, the first detection probability would be reduced by broad barriers. This represents a trade-off between acceleration of evolution and detection.

## 4. Asymptotic Results

In the case of ϵ=0, the decays of the long time limit of the detection probability show different behaviors for different initial conditions: the oscillating probability, which generally emerges in quantum mechanics, and the monotonous probability, which occurs in classical mechanics (see Figure 2).

In this section, we address the asymptotic formula to discuss when the first detection probability oscillates for a small sampling time τ. Previously, Schrödinger studied the first arrival time of Brownian motion, namely, the limit of the classical random walk [55]. He demonstrated that the probability density function was a fat tail and decayed with the power law −3/2. In [32], the large *n* limit of $\varphi_{n}$ was obtained for a single source, namely, an initial condition on one node of the graph. The behavior of $\varphi_{n}$ exhibits a power-law decay with oscillations, which is quite different from its classical counterpart, which decays monotonically.

In Equation (23), the Bessel function can be expanded as an asymptotic formula for large arguments [36]:

(30) $$J_{\mu }\left(x\right)∼\sqrt{\frac{2}{\pi x}}\cos\left(x-\frac{\mu \pi }{2}-\frac{\pi }{4}\right).$$

Due to the linearity of quantum mechanics, all we need to do to consider a Gaussian initial condition is to sum over the different amplitudes with the weights $c_{j}$. Therefore, the amplitude of the first detection for the Gaussian initial wave packet is

(31) $$\begin{array}{cc} \varphi_{n}∼ & \sum_{j}c_{j}\sqrt{\frac{\tau }{\pi n^{3}}}\left\{e^{i(2n\tau +\frac{\pi }{4})}\left(\frac{1+\delta_{j_{d}-j,0}}{2}-\frac{i}{\pi }\int_{0}^{\pi }dk\frac{\sin^{2}\frac{k(j_{d}-j)}{2}}{\tan(2\tau \sin^{2}\frac{k}{2})}\right)\right.\\ \; & \left.+e^{-i(2n\tau +\frac{\pi }{4})}\left(\frac{{\left(-1\right)}^{j_{d}-j}+\delta_{j_{d}-j,0}}{2}+\frac{i}{2\pi }\int_{0}^{\pi }dk\frac{{\left(-1\right)}^{j_{d}-j}-\cos k\left(j_{d}-j\right)}{\tan[\tau (1+\cos k)]}\right)\right\}. \end{array}$$

Notice the $n^{-3/2}$ over all decay of the first detection amplitude, and recall that this is similar to the classical result. When squared, $F_{n}$ will be proportional to $n^{-3}$, with possible superpositions of oscillations which depend on parameters such as the width. Then, Equation (31) can be abbreviated with the auxiliary function r(ν,τ) and β(ν,τ) [32]:

(32) $$\varphi_{n}∼\sqrt{\frac{4\tau }{\pi n^{3}}}\sum_{j}c_{j}r\left(j_{d}-j,\tau \right){\mathrm{trig}}_{j_{d}-j}\left[2n\tau +\frac{\pi }{4}+\beta \left(j_{d}-j,\tau \right)\right].$$

The auxiliary function satisfies the relation [32]

(33) $$r\left(\nu ,\tau \right)e^{i\beta (\nu ,\tau )}=\frac{1+\delta_{\nu ,0}}{2}-\frac{i}{\pi }\int_{0}^{\pi }\frac{\sin^{2}\left(\nu k/2\right)}{\tan[2\tau \sin^{2}\left(k/2\right)]}dk,$$

and the trigonometric function is

(34) $${\mathrm{trig}}_{\mu }\left(x\right)=\frac{e^{ix}+{\left(-1\right)}^{\mu }e^{-ix}}{2}.$$

In the limit of small sampling time τ [32],

(35) $$r\left(\mu ,\tau \right)e^{i\beta (\mu ,\tau )}∼-i\frac{\mu }{2\tau },$$

thus, the first detection amplitude can be written as

(36) $$\varphi_{n}∼\sqrt{\frac{1}{4\pi \tau n^{3}}}\sum_{j}ic_{j}\left(j_{d}-j\right){\left(-1\right)}^{j_{d}-j}e^{-i(2\tau n+\frac{\pi }{4})}-\sqrt{\frac{1}{4\pi \tau n^{3}}}\sum_{j}ic_{j}\left(j_{d}-j\right)e^{i(2\tau n+\frac{\pi }{4})}.$$

Note that the periodicity is determined by 2τn≃2π. We assume that the detection site and the center of the initial wave packet are situated on even lattice points, and find

(37) $$\begin{array}{cc} \varphi_{n} & ∼\sqrt{\frac{1}{\pi \tau n^{3}}}\left[\sum_{j_{even}}c_{j}\left(j_{d}-j\right)\sin\left(2\tau n+\frac{\pi }{4}\right)-i\sum_{j_{odd}}c_{j}\left(j_{d}-j\right)\cos\left(2\tau n+\frac{\pi }{4}\right)\right]\\ \; & =\sqrt{\frac{1}{\pi \tau n^{3}}}\left[\sum_{j_{even}}\frac{1}{\sqrt{A}}e^{\frac{-{\left(j-j_{c}\right)}^{2}}{4\sigma^{2}}}\left(j_{d}-j\right)\sin\left(2\tau n+\frac{\pi }{4}\right)-\sum_{j_{odd}}\frac{i}{\sqrt{A}}e^{\frac{-{\left(j-j_{c}\right)}^{2}}{4\sigma^{2}}}\left(j_{d}-j\right)\cos\left(2\tau n+\frac{\pi }{4}\right)\right]. \end{array}$$

Due to the symmetry of the wave packet, the prefactor of the trigonometric function of the odd part can be written as

(38) $$\sum_{j_{odd}}c_{j}\left(j_{d}-j\right)=\sum_{\kappa =1,3,5,\cdots }\frac{1}{\sqrt{A}}e^{\frac{-\kappa^{2}}{4\sigma^{2}}}\left(2j_{d}-2j_{c}\right)=\frac{1}{\sqrt{A}}\left(j_{d}-j_{c}\right)\vartheta_{2}\left(0,e^{-\frac{1}{\sigma^{2}}}\right),$$

and the even part is

(39) $$\begin{array}{cc} \sum_{j_{even}}c_{j}\left(j_{d}-j\right) & =\sum_{\lambda =0,2,4,\cdots }\left[\frac{1}{\sqrt{A}}e^{-\frac{\lambda^{2}}{4\sigma^{2}}}\left(2j_{d}-2j_{c}\right)\right]-\frac{1}{\sqrt{A}}\left(j_{d}-j_{c}\right)\\ \; & =\frac{1}{\sqrt{A}}\left(j_{d}-j_{c}\right)\vartheta_{3}\left(0,e^{-\frac{1}{\sigma^{2}}}\right), \end{array}$$

where $\vartheta_{\nu }\left(z,q\right)$ is the Elliptic Theta function [56].

When σ<1, the second-order Elliptic Theta function is not equal to the third-order one, and the trigonometric function in the first detection probability stays alive; consequently, $F_{n}$ decays with added oscillations when σ<1.

When σ≥1, the two elliptic functions behave similarly, that is,

(40) $$\sum_{j_{odd}}c_{j}\left(j_{d}-j\right)=\sum_{j_{even}}c_{j}\left(j_{d}-j\right).$$

Thus, the trigonometric function vanishes and the first detection amplitude is

(41) $$\varphi_{n}∼\sqrt{\frac{1}{\pi \tau n^{3}}}\frac{1}{\sqrt{A}}\left(j_{d}-j_{c}\right)\vartheta_{3}\left(0,e^{-\frac{1}{\sigma^{2}}}\right)e^{i(2\tau n-\frac{\pi }{4})}.$$

In the limit of large *n*, the first detection probability for σ≥1 is

(42) $$\begin{array}{cc} F_{n}∼{\left|\varphi_{n}\right|}^{2} & =\frac{{\left(j_{d}-j_{c}\right)}^{2}\vartheta_{3}{\left(0,e^{-1/\sigma^{2}}\right)}^{2}}{\pi \tau n^{3}A}\\ \; & =\frac{{\left(j_{d}-j_{c}\right)}^{2}\vartheta_{3}{\left(0,e^{-1/\sigma^{2}}\right)}^{2}}{\sqrt{2\pi^{3}}\sigma \tau n^{3}\vartheta_{3}\left(-j_{c}\pi ,e^{-2\sigma^{2}\pi^{2}}\right)}. \end{array}$$

**Figure 8:**
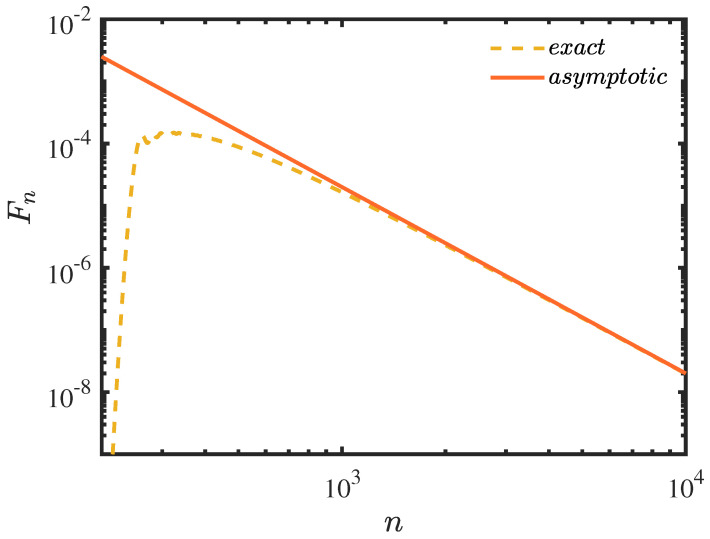
The plot of the asymptotic result of Equation (42) (log−log). The width of the Gaussian wave is σ=1, the detection state $|\psi_{det}\rangle =|90\rangle$, the initial state is centred at $j_{c}=-10$, and the sampling time τ=0.2.

In Figure 8, the asymptotic Equation (42) fits well with the exact result, and the first detection probability decays monotonically with the power-law −3. The prefactor is related to the width of the wave packet σ and the distance between the center of the wave packet $j_{c}$ and the detection site $j_{d}$. When the width is larger than the lattice constant, which in this paper is 1, the probability decays monotonically with the power-law −3 in the absence of oscillations present for narrower initial states.

## 5. Conclusions and Discussions

We have investigated the first detection problems of a continuous-time quantum walk on an infinite (im)pure lattice under the repeated measurement. The impurity in the lattice is described as one of the diagonal elements of the Hamiltonian. We show the relation between the decay of the first detection probability and the width of the initial Gaussian wave. We found that the distribution of the first detection probability decays while oscillating with the power-law −3 for the initial narrow wave, i.e., σ<1. Conversely, the distribution decays monotonically, as Schrödinger described when he considered the first passage time of the Brownian motion. In the limit of large *n*, as Equation (42) shows, the probability is sensitive to the distance between the detection site $j_{d}$ and the center of the initial wave packet $j_{c}$. For an impure lattice, the walker tunnels through the barrier with different transmission probabilities which depend on the components of the momentum of the initial wave. For large σ, i.e., corresponding to a single ingredient in momentum space, the probability keeps decaying monotonically when the walker goes back and forth between the barrier and the detection site. By comparing the incident time $t_{in}$ between the barrier case and the pure system, we found that the incident time in the impure system is smaller than the pure one, and is strongly sensitive to the width of the wave. In other words, the evolution of the walker speeds up. Furthermore, the total probability of detection $P_{tot}$ is always less than unity, and there exits an optimal value in the vicinity of the critical sampling time τ=mτ/2. However, the critical point is transformed by the presence of the barrier and the period is destroyed.

By tuning the width of the initial wave and the distance between the detector and the center of the wave, it is possible to succeed in detecting the particle more efficiently. Defects and the nature of the materials can accelerate successful detection or reduce the probability of successful detection. We hope that our results for the stroboscopically probed system can help in optimizing the read-out of the state of a quantum computer.

## Data Availability

The data presented in this study are available on request from the corresponding author. The data are not publicly available due to privacy.

## Abbreviations

The following abbreviations are used in this manuscript:

QW	Quantum Walk
CTQW	Continuous-Time Quantum Walk
DTQW	Discrete-Time Quantum Walk

## Appendix A

### Appendix A.1

In this appendix, we present the Green Function for a tight-binding Hamiltonian with a single impurity. Here, the Hamiltonian of the impurity system is

(A1) $$\begin{array}{cc} H & =H_{0}+H_{1}\\ \; & =-\sum_{j}(|j\rangle \langle j+1|+|j+1\rangle \langle j|)+\epsilon |0\rangle \langle 0|, \end{array}$$

while the eigenvalues and eigenfuctions of $H_{0}$ have been exhibited in the main text,

(A2) $$E_{k}=-2\cos k,\;|\psi_{k}\rangle =\frac{1}{\sqrt{2\pi }}\sum_{j}e^{ikj}|j\rangle .$$

The Green function corresponding to the Schrödinger equation satisfies

(A3) $$\left[E-H(l)\right]G\left(l,l^{\prime };E\right)=\delta \left(l-l^{\prime }\right),$$

and the solution of the aforesaid equation is

(A4) $$\begin{array}{cc} G & =G_{0}+G_{0}TG_{0}\\ \; & =G_{0}+G_{0}|0\rangle \frac{\epsilon }{1-\epsilon G_{0}\left(0,0\right)}\langle 0|G_{0}, \end{array}$$

where $G_{0}\left(0,0\right)=\langle 0|G_{0}|0\rangle$, $G_{0}$ is the Green function of the non-perturbed Hamiltonian. The poles of *G* correspond to the discrete eigenvalues (bound state) of *H*. In the present case, the eigenvalue of the bound state is provided by

(A5) $$G_{0}\left(0,0,E_{b}\right)=\frac{1}{\epsilon },$$

and the bound state is provided by

(A6) $$|\psi_{b}\rangle =\sum_{j}b_{j}|j\rangle ;\;b_{j}=\frac{G_{0}\left(j,0,E_{b}\right)}{\sqrt{-G_{0}^{\prime }\left(0,0,E_{b}\right)}},$$

where the prime denotes differentiation with respect to *E* and the Green function is as follows:

(A7) $$G_{0}\left(l,m,z\right)=\frac{{\left(-\frac{z}{2}+\sqrt{\frac{z^{2}}{4}-1}\right)}^{\left|l-m\right|}}{\sqrt{z^{2}-4}}.$$

The eigenstates in the energy band are scattering states $\left(|\psi_{s}\rangle \right)$, and can be written as

(A8) $$|\psi_{s}\rangle =|\psi_{k}\rangle +G_{0}^{+}\left(E_{k}\right)T^{+}\left(E_{k}\right)|\psi_{k}\rangle .$$

Therefore, the *j*-site amplitude of $\psi_{s}$ is

(A9) $$\langle j|\psi_{s}\rangle =\langle j|\psi_{k}\rangle +\frac{\langle j|G_{0}^{+}\left(E_{k}\right)|0\rangle \epsilon \langle 0|\psi_{k}\rangle }{1-\epsilon G_{0}^{+}\left(0,0,E_{k}\right)},$$

where the matrix elements are

(A10) $$\langle l|G_{0}^{+}\left(E_{k}\right)|m\rangle =\frac{-i}{\sqrt{4-E_{k}^{2}}}{\left(-\frac{E_{k}}{2}+i\sqrt{1-\frac{E_{k}^{2}}{4}}\right)}^{\left|l-m\right|}.$$

With the eigenfunctions from Equations (A6) and (A9), we can obtain the propagator $\langle j_{2}\left|U(n\tau )\right|j_{1}\rangle$ as in the main text.
